# The financial burden of SARS-CoV-2 pregnancies in a tertiary exclusive COVID-19 maternity

**DOI:** 10.25122/jml-2024-0128

**Published:** 2024-05

**Authors:** Tina-Ioana Bobei, Romina-Marina Sima, Gabriel-Petre Gorecki, Mihaela Amza, Anca Bobircă, Mihai Popescu, Bashar Haj Hamoud, Liana Pleș

**Affiliations:** 1 Department of PhD Studies, IOSUD, Carol Davila University of Medicine and Pharmacy, Bucharest, Romania; 2 Department of Obstetrics and Gynecology, Carol Davila University of Medicine and Pharmacy, Bucharest, Romania; 3 Bucur Maternity, Saint John Hospital, Bucharest, Romania; 4 Faculty of Medicine, Titu Maiorescu University, Bucharest, Romania; 5 Department of Anesthesiology, CF2 Hospital, Bucharest, Romania; 6 Department of Internal Medicine and Rheumatology, Carol Davila University of Medicine and Pharmacy, Bucharest, Romania; 7 Department of Anesthesia and Critical Care, Carol Davila University of Medicine and Pharmacy, Bucharest, Romania; 8 Department of Gynecology, Obstetrics and Reproductive Medicine, Saarland University Hospital, Homburg, Germany

**Keywords:** COVID-19, SARS-COV-2, pregnancy

## Abstract

The COVID-19 pandemic had a major impact on health systems worldwide, and Romania was no exception. The impact on healthcare expenses for pregnant women was considerable, especially in COVID-19-only tertiary centers. This study aimed to analyze the impact of the COVID-19 pandemic on healthcare costs in a designated COVID-19 maternity ward. We conducted an observational study comparing pregnant women with SARS-CoV-2 (study group) to those without the infection (control group). Patients were recruited at Bucur Maternity Hospital from March 2020 to March 2022. We evaluated expenses for the entire period of hospitalization, treatment, medical supplies, and medical investigations. The study included 600 pregnant women, divided equally into two groups of 300 each. Significant cost differences were observed between the COVID-19 and non-COVID-19 groups: medication costs (664.56 EUR vs. 39.49 EUR), administrative costs (191.79 EUR vs. 30.28 EUR), and medical investigation costs (191.15 EUR vs. 29.42 EUR). The costs for a severe case of COVID-19 were about two times higher than a mild case and 70 times higher than a non-COVID-19 case (*P* <0.001). We identified a significant cost increase due to SARS-CoV-2 infection in our unit. The expenses were augmented by the time of hospitalization, medication, and medical investigations. COVID-19 had a significant impact on healthcare costs, mostly among pregnant women with severe disease. The strategy of operating exclusively as a COVID-19 unit proved to be inefficient and highly costly to our hospital.

## INTRODUCTION

Since its discovery in November 2019, Severe Acute Respiratory Syndrome Coronavirus 2 (SARS-CoV-2) infected an estimated 200 million people around the world and has been responsible for more than 4 million deaths worldwide [1]. As the virus rapidly spread across continents, the World Health Organization (WHO) declared COVID-19 a global health emergency in March 2020 [2]. The WHO emphasized the importance of early identification, isolation, and management of patients, along with contact tracing and social distancing measures to interrupt the transmission chain [3].

The development of COVID-19 disease, the increasing number of patients, and the complications of the disease have imposed high medical costs on both patients and the healthcare system [4]. These costs vary based on several factors, such as the number of infections, disease severity, the average length of hospitalization, and the average time spent in intensive care units [5,6]. Studies have shown that the economic burden of direct medical costs for patients with COVID-19 was significantly higher than for other infectious diseases due to higher mortality and a higher possibility of hospitalization [7].

The COVID-19 pandemic significantly impacted health systems worldwide, and Romania is no exception. Our country's health system was already dealing with many difficulties before the pandemic, including a lack of medical staff, equipment, and funding [8]. The pandemic exacerbated these issues and underscored the urgent need for health system reform in Romania [9]. There was no specific treatment for SARS-COV-2, and tremendous efforts were made to find an effective treatment to reduce morbidity and mortality [10].

The impact on healthcare expenditure for pregnant women was considerable, especially in tertiary centers destined for COVID-19 cases. The increased demand for healthcare services during the pandemic strained the health system, leading to higher expenditures [11]. A significant factor contributing to rising healthcare costs was the need for personal protective equipment (PPE) for healthcare workers [11]. The cost of PPE increased due to increased demand and lack of availability, which resulted in more significant healthcare costs [12]. In addition, many hospitals implemented additional safety measures, such as increased tests for SARS-CoV-2 and isolation protocols for pregnant women. Those measures resulted in longer hospital stays and additional medical procedures, increasing healthcare costs [5].

This study aimed to analyze the impact of SARS-CoV-2 infection on healthcare costs by examining case management in our COVID-only maternity unit.

## MATERIAL AND METHODS

The objective of the study was to analyze if the model adopted by the national health system of completely separating obstetrical care for COVID-19 and non-COVID-19 patients had a significant impact in terms of costs for our hospital. We conducted an observational study where we compared a group of pregnant women infected with SARS-CoV-2 (study group) with a control group of uninfected pregnant women.

Patients were recruited from Bucur Maternity, Sf. Ioan Hospital, Bucharest. This maternity is uniquely situated 3 km away from the main hospital. On March 19, 2020, following a decision by the Ministry of Health, it was designated as a COVID-19 tertiary maternity. Thus, Bucur Maternity exclusively treated SARS-CoV-2-positive obstetrical or gynecological patients. This was part of a broader strategy in Romania, where various medical facilities were converted into COVID-19-only units.

Our department of obstetrics typically handles approximately 2000-2200 births per year. During the period when Bucur Maternity exclusively treated COVID-19 patients, we conducted over 2400 evaluations in the emergency department and facilitated 535 births for COVID-19 patients. Bucur Maternity had specific protocols during the pandemic regarding the admission and discharge of patients. From March 19, 2020, to March 12, 2022, Bucur Maternity admitted exclusively COVID-19 patients, except from July 1, 2021, to October 1, 2021, when both COVID-19 and non-COVID-19 were admitted based on Ministry of Health directives.

Admission criteria for SARS-CoV-2-positive pregnant women varied throughout the pandemic. Initially, all pregnant women testing positive via antigen or PCR were hospitalized, regardless of symptoms, and were discharged only after testing negative, leading to hospital stays of 3-4 weeks. Later, protocols changed to discharge patients 14 days after the first positive test. From 2021, only symptomatic patients were admitted, and during the vaccination period, vaccinated patients were discharged 5 days after symptom onset, while unvaccinated patients were discharged 7 days after symptom onset.

The patients in the study were recruited from March 2020 to March 2022 after signing the informed consent. Inclusion criteria for the study group were as follows: pregnant women with a positive RT-PCR or antigen test for SARS-CoV-2, gestational age between 24 and 40 weeks, live fetus, singleton pregnancies, hospitalization for a minimum of 24 hours, and delivery at our unit. To assess the impact of SARS-CoV-2 infection on costs, we compared the data from patients with COVID-19 with a control group of 300 pregnant women who gave birth at Bucur Maternity before the pandemic, specifically between January 2019 and January 2020. The inclusion criteria for the control group were similar: gestational age between 24 and 40 weeks, live fetus, singleton pregnancies, and delivery at our unit.

Exclusion criteria for both groups included age under 16 years old, refusal to participate, twin pregnancies, pregnant women admitted for obstetrical conditions who did not give birth before discharge, patients discharged before 24 hours, and stillbirth.

The parameters studied included maternal age, gestational age, type of SARS-CoV-2 test, number of days from infection, type of birth, presence of symptoms (cough, fever, chills, shortness of breath, and other uncommon symptoms), severity of infection, antiviral treatment, number of intensive care unit (ICU) admission days, total hospitalization days, expenses related to hospitalization, treatment, medical supplies, and blood tests. All data were obtained from the patient's electronic register.

All cost information was extracted from the electronic records of our hospital. Upon discharge, each patient received a detailed cost report (not a bill, as this is a public hospital with no patient fees) outlining the total and separate costs for hospitalization, medical supplies, medications, and additional expenses such as medical equipment.

Data analysis was performed using the Statistical Package for the Social Sciences (SPSS) v. 19.0. We analyzed the characteristics of all patients using descriptive statistic tests of each group separately. Depending on the data format, we employed tests such as the t-test, chi-square test, Mann–Whitney U test, non-parametric tests, and Pearson’s correlation. *P* values of <0.05 were considered statistically significant.

## RESULTS

**Figure 1:**
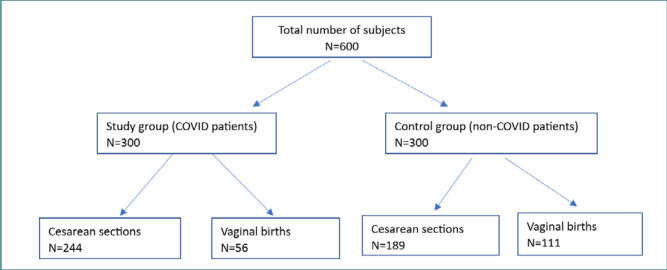
Patient distribution in the study

The study included 600 pregnant women who gave birth at our maternity, divided into 300 patients positive for SARS-CoV-2 (study group) and 300 patients negative for SARS-CoV-2 (control group). The distribution is illustrated in Figure 1.

We analyzed the general data of each group, and we observed that in the study group, the mean maternal age was 30.58 years compared to 27.36 years in the control group. During the COVID-19 period, 10% of patients were hospitalized with pregnancies under 37 weeks of gestation, compared to 4.62% in the non-COVID-19 group. Significant differences were identified, particularly in the type of birth, with an increased incidence of cesarean births during the COVID-19 period (81.36% vs. 63.32%), as shown in Table 1.

**Table 1:** General data of the patients

Parameters	COVID-19 group (*n* = 300)	Non-COVID-19 group (*n* = 300)	*P* value
**Maternal age (years, ranges)**	30.58 (18–47)	27.36 (18–39)	<0.001
**Gestational age (%)**	≥ 37 weeks (90%)	≥ 37 weeks (95.38%)	<0.001
	< 37 weeks (10%)	< 37 weeks (4.62%)	
**Type of birth (vaginal vs. cesarean, %)**	Vaginal birth (18.64%)	Vaginal birth (36.68%)	<0.001
	Cesarean birth (81.36%)	Cesarean birth (63.32%)	
**Hospitalization days (mean, ranges)**	6.1 (1–26)	4.79 (1–11)	<0.001
**Total expense (EUR, mean, ranges)**	3422.3(673–31007.57)	1990.8 (419.66– 12088.04)	<0.001
**Hospitalization expense (EUR, mean, ranges)**	10281.18 (2141.91–10523.13)	1767.60(272.80–5066.69)	<0.001
**Medicines expense (EUR, mean, ranges)**	664.56 (7.16–4438.63)	39.49 (2.47 – 399.84)	<0.001
**Medical supplies (EUR, mean, ranges)**	191.79 (8.12– 996.21)	30.28 (3.75–355.26)	<0.001

The average length of hospitalization for the COVID-19 group was 6.1 days, higher than the 4.79 days observed in the non-COVID-19 period. We converted all costs from the national currency, the new Romanian leu (RON), to Euro (EUR) at an exchange rate of 1 EUR = 4.8 RON to better understand the costs worldwide.

The most important differences appear in medication costs (664.56 EUR vs. 39.49 EUR), especially in terms of antivirals, medical investigations such as blood tests, computerized tomography (CT), and thoracic radiography (166.78 EUR vs. 27.43 EUR) or in the costs of sanitary materials (191.79 EUR vs. 30.28 EUR). The increase was also determined by introducing compulsory equipment consisting of overalls or reinforced gowns and FFP2 or FFP3 masks. The cost of medical investigations also increased by six times because of a long list of blood tests required to determine the appropriate treatment, which was repeated to monitor patients' evolution, such as chest X-rays and CT scans, which were often necessary.

We also identified the costs based on the mode of delivery and the significant differences between cesarean or vaginal births within the study and control group (Table 2). In both groups, vaginal births had lower costs in terms of hospitalization, medical supplies, or investigations. The differences between the two groups were statistically significant (*P* <0.005).

**Table 2:** Costs based on mode of delivery

Parameters	COVID-19 group (*n* = 300)		Non-COVID-19 group (*n* = 300)	
**Type of birth**	Cesarean section	Vaginal birth	Cesarean section	Vaginal birth
**Hospitalization expense** **(EUR, mean, ranges)**	2165.30 (672.92- 17157.05)	2010.56 (777.89-3864.46)	1824.99 (571.15- 3897.45)	811.87 (272.80-4287.20)
**Medicines expense** **(EUR, mean, ranges)**	724.54 (76.38- 10523.13)	192.08 (7.38- 1891.29)	55.71 (24.71- 399.84)	15.16 (2.47- 50.95)
**Medical supplies (EUR, mean, ranges)**	211.12 (60.28- 4438.63)	111.25 (8.12-356.74)	31.76 (12.15- 100.07)	17.92 (3.75- 67.06)
**Medical investigations** **(EUR, mean, ranges)**	191.15 (51.43- 1601.16)	67.10 (9.92- 110.22)	29.42 (2.53- 168.49)	21.47 (6.5-112.33)
**Total expense** **(EUR, mean, ranges)**	3314.68 (881.31- 17157.04)	2393.86 (1266.48- 4431.42)	1957.37 (837.38- 4025.55)	1805.31 (419.66-1805.31)

We found a significant increase in all costs for SARS-CoV-2-positive patients. Table 3 highlights the factors contributing to high expenses during the pandemic period.

**Table 3:** Costs differences

	COVID-19 group (*n* = 300)	Non-COVID-19 group (*n* = 300)	*P* value
**Antibiotherapy (%)**	97.5%	63.3 %	<0.001
**Corticotherapy (%)**	66.7%	3.9 %	<0.001
**Antivirals (%)**	8.3%	-	
**Intensive care unit days (mean, ranges)**	2.18 (0-15)	0.68 (0-1)	<0.001
**Thoracic radiography (%)**	63.3%	0.12 %	<0.001
**Sets of blood tests per patient (%)**	1 set (36.7%)	1 set (63.4%)	<0.001
	2 sets (53.5%)	2 sets (33.2%)	
	≥3 sets (9.8 %)	≥3 sets (3.4%)	

Medication costs increased due to higher use of antibiotics (97.5% vs. 63.3%), corticosteroids (66.7%), and antivirals (8.3%). Table 4 illustrates the distribution of SARS-CoV-2 patients according to vaccination status, disease severity, and outcomes during hospitalization. In the study group, 16.8% of patients were vaccinated against COVID-19. Among these patients, 32.2% experienced moderate to severe forms of the disease. Additionally, 77.5% had a favorable outcome during hospitalization, while 19.8% required transfer to another facility.

**Table 4:** COVID-19 characteristics

Days of infection (mean, ranges)	4.51 (1-26)	
**Vaccination status (%)**	Unvaccinated	83.6 %
	Vaccinated	16.8 %
**Severity (%)**	Asymptomatic and mild	67.9 %
	Moderate	14.3 %
	Severe	17.9 %
**Patient evolution (%)**	Improved	77.5%
	Transfer	19.8 %
	Dead	2.7%

The average length of ICU hospitalization for patients with COVID-19 was 2.18 days, with a maximum of 15 days, compared to 0.68 days for non-COVID cases. Severe COVID-19 cases (17.9%) required ICU stays of more than 5 days, often necessitating mechanical ventilation.

An increase in the number of investigations per patient for pregnant women with COVID-19 can explain the rising costs noted in Table 1. Table 3 reveals a higher percentage of chest X-rays (63.3%) performed routinely in moderate and severe cases of COVID-19, as well as an increase in the number of blood investigations per patient (1 set – 36.7% vs. 63.4%; 2 sets – 53.5% vs 33.2%; ≥ 3 sets – 9.8% vs. 3.4%).

**Figure 2:**
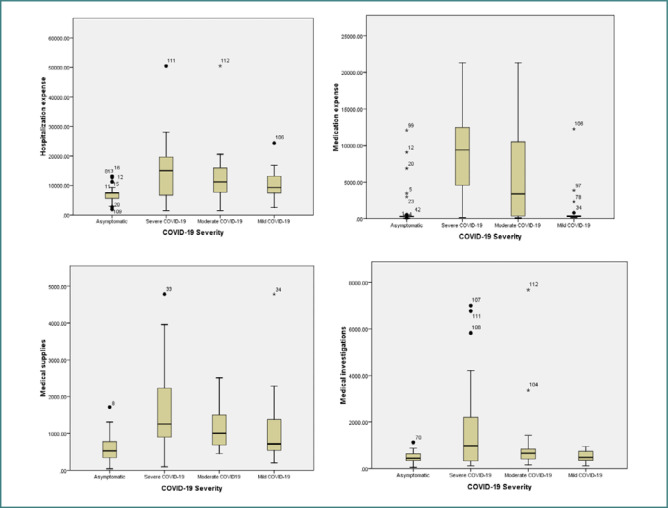
Distribution of cost components by COVID-19 severity

Figure 2 represents the distribution of cost compounds (hospitalization, medication, medical supplies, and medical investigations) for pregnant women with COVID-19 according to disease severity in a more schematic form.

Costs increased significantly with the presence and severity of infection. There was a strong correlation between case severity and direct and indirect hospitalization costs (*P* <0.001). The costs of a severe case of COVID-19 were about two times higher than a mild case and 70 times higher than a non-COVID-19 case.

To reduce the risk of bias, we analyzed the comorbidities within the two groups as labeled in Table 5. The impact of comorbidities on costs was not significant because both groups were homogenous regarding associated pathologies (*P* <0.005). Even though pregnant women in our study were infected with SARS-CoV-2, they were representative of the general female population, which is typically not characterized by preexisting comorbidities. This was also true for patients without COVID-19 who gave birth.

**Table 5:** Comorbidities across study groups

Comorbidities	COVID-19 group (*n* = 300)	Non-COVID-19 group (*n* = 300)	*P* value
**Obesity (%)**	3.5%	4.2%	<0.005
**Gestational diabetes (%)**	1.2%	0.8%	<0.005
**Gestational hypertension (HTA, %)**	2.3%	1.9%	<0.005
**Anemia (%)**	33.4%	25.7%	<0.005

## DISCUSSION

The objective of this study was to highlight the impact of SARS-CoV-2 infection on healthcare system resources through the perspective of a maternity unit exclusively designated for patients with COVID-19. We demonstrated a significant increase in the costs of hospitalization, medication, and investigations correlated with the increase in the severity of the disease, but also compared to the cases of pregnant women without COVID-19.

The increase in costs was due to several factors, such as an increase in the percentage of cesarean births, premature births, the number of days spent in ICU, multiple medications (antibiotics, corticosteroids, antivirals), and routine investigations in moderate and severe cases of illness (chest X-rays, chest CT, multiple sets of blood tests). In other studies, we also found increased cesarean and premature births during the pandemic [13-16]. In the pre-COVID-19 period, the median length of hospitalization in our hospital was 4.79 days, with a total cost of approximately 415 EUR/day of hospitalization. Our findings suggest that pregnant women positive for SARS-CoV-2 infection, on average, stayed in the hospital for about 6.1 days, at an approximate cost of 560 EUR/ day of hospitalization, with significant differences in both cesarean births (2165.30 EUR vs. 1824.99 EUR, and especially vaginal births (2010.56 EUR vs. 811.87 EUR). The main determinant of this increase in cost is related to medications, PPE, or hospitalization time needed to care for patients with COVID-19.

A 2020 review reported that the median cost of a vaginal delivery was $40 in public hospitals, while a cesarean delivery cost was $178 in low-income and middle-income countries before the SARS-CoV-2 pandemic. For pregnant women with COVID-19, these costs increased sixfold for vaginal births and sixteenfold for cesarean births [17]. Our findings suggest that the state incurs twice the cost for vaginal births and up to seventy times the cost for severe COVID-19 cases in cesarean births. This finding mainly concerns public hospitals, foundational for achieving universal health coverage [18].

It has been ascertained that tertiary hospitals are significantly more expensive than secondary and primary health units, mainly because of the specialist skills concentrated there [19]. A major cost driver specific to the pandemic has been the surge in PPE needed to care for COVID-19 patients. This surge in PPE as a major cost driver (up to 50%) is worrying. Previously, while cost factors varied by country, most reported that medicines and supplies, transport, and accommodation were the main cost factors women had to address to access care [18]. Another emerging cost factor is medical oxygen, accounting for up to 48% of costs in severe cases requiring prolonged hospital stays. This is particularly concerning given its status as the second most crucial element in COVID-19 treatment [19].

Before the pandemic, PPE use was limited for specific conditions for healthcare providers. However, COVID-19 demanded comprehensive protection, leading to a global surge in demand that quickly exceeded supplies worldwide [20]. More than half of healthcare workers reported inadequate access to PPE for safe consultations [21]. At the beginning of the pandemic, the cost of PPE was lower, but it skyrocketed, along with the price of surgical masks (six times), N95 respirators (three times), and surgical gowns (two times) [20]. The high costs of caring for pregnant women with COVID-19 underscores the importance of preventive measures like vaccination, which would have substantial cost-saving implications [22]. In our study, only 16.8% of pregnant women were vaccinated at admission. Unfortunately, the vaccination campaign has not been very successful since its launch in October 2021, and all pregnant women with severe COVID-19 were unvaccinated in the present study.

There are multiple studies about the impact of the pandemic on healthcare costs in which increased use of healthcare resources and a significant increase in costs were found. It is challenging to compare expenditures because of differences in healthcare costs, population, or methodology. There are studies from Switzerland [23], USA [24], Turkey [25,26], China [27], and Saudi Arabia [28] where the burden on the healthcare system in terms of resource use and costs is substantial. In a study of 70 patients in China during the early period of the pandemic, the cost per patient was identified as 6,827 USD, with medication constituting the largest proportion at nearly 45.1% [19]. Similarly, a study in Italy revealed that treating a single COVID-19 patient in their hospitals costs the state almost €8,500, with even higher expenses, reaching nearly €9,800 for each patient who died from COVID-19 in hospital [29]. These findings underscore the substantial impact of the pandemic on healthcare spending worldwide, as evidenced by other studies [30,31]. Although existing research explores the financial burden of COVID-19 treatment in general hospitals, a critical gap remains in our understanding of costs associated specifically with maternity care during the pandemic. This is an element of originality in our study, given the national medical context that designated our hospital as a COVID-19 tertiary unit for pregnant women.

One limitation of our study is the heterogeneity of admission criteria from the beginning to the end of the pandemic, significantly influencing costs. Moreover, the discharge criteria and the SARS-COV-2 testing also changed and impacted the costs. Secondly, rapid transfers of patients from our department may have biased our data on hospitalization lengths during the pandemic.

While previous research suggests a positive correlation between healthcare spending and economic growth [32], the COVID-19 pandemic presents a contrasting scenario [33]. These effects extend far beyond the health costs. The International Monetary Fund estimates the cost of COVID-19 to be three trillion euros for the European Union [34].

This study attempted to estimate the COVID-19 burden in COVID-19-only centers based on data from a national COVID-19 tertiary unit for pregnant women. A key aspect of our investigation is the cost implication of the extensive safety measures implemented to protect healthcare workers during patient interactions. These measures include personal protective gowns, gloves, and masks such as N95, FFP2, and FFP3, as well as face shields and goggles. Efforts such as converting existing areas or creating special wards isolated from other parts of the hospital and dividing spaces into 'red' zones for infected patients and 'green' zones for medical staff also generated additional costs. In addition, performing COVID-19 diagnostic tests on every patient or newborn, repeated at certain times, especially during the first wave, increased care costs for other diseases.

In the early stages of the COVID-19 pandemic, doctors hospitalized patients for close monitoring regardless of the severity of their symptoms. Over time, this approach shifted, and only patients with more severe symptoms or lower oxygen saturation levels were hospitalized. In September 2020, the WHO recommended outpatient monitoring for patients with mild symptoms [35].

The economic impact of the pandemic on hospitals that had to limit access to health services for non-COVID patients needing medical care is another issue requiring further investigation. COVID-19 has significantly increased expenditure in the Romanian health system: 594 million euros were allocated to the National Health Insurance House to cover the cost of anti-COVID treatments in hospitals, and 214 million euros were spent on purchasing equipment and paying bonuses to doctors. This money was budgeted for the purchase of protective equipment, payment of bonuses to medical staff, tests, and expansion of health services accessible to vulnerable groups, but so far, there are no reported settlements.

The financial support of healthcare facilities during a pandemic can be improved by introducing a combination of strategies to ensure adequate funding. Some approaches would involve allocating additional funds specifically for healthcare facilities to manage increased patient numbers and purchase necessary equipment or for facilities serving vulnerable populations or those in underserved areas and ensuring proper reimbursement by the national insurance fund for COVID-19-related expenses, including testing, treatment, and care delivery. Additionally, funds should be allocated for telemedicine services, covering infrastructure development, technology upgrades, and training of health professionals. Telemedicine can help healthcare institutions reach patients remotely and reduce pressure on physical infrastructure.

Anticipating COVID-19-related complications and identifying high-risk subgroups can be achieved by performing epidemiologic data analysis to uncover patterns and trends. Factors such as age, comorbidities, geographic location, and socioeconomic status may be correlated with an increased risk of complications. Similarly, reviewing clinical data can reveal risk factors and predictors of severe COVID-19 outcomes, while monitoring biomarkers and laboratory parameters can help predict disease progression and identify patients at higher risk of complications. For example, elevated inflammatory markers such as C-reactive protein (CRP) and procalcitonin have been associated with severe COVID-19 outcomes [36]. In addition, interdisciplinary collaboration between epidemiologists, clinicians, data scientists, and public health experts is critical for effectively using predictive analytics in pandemic response efforts.

## CONCLUSION

We observed a significant cost increase across all categories in our unit due to SARS-CoV-2 infection. These expenses increased due to extended hospital stays, increased medication use, and more frequent medical investigations. Patients with severe forms of the disease incurred costs that were 70 times higher than those without COVID-19 complications. Additionally, the strategy of operating a COVID-exclusive maternity ward proved to be inefficient and costly, particularly as it reduced access to obstetrical prenatal care for non-COVID patients. To assess the broader financial impact on our national healthcare system, conducting a multicentric study comparing the costs in exclusively COVID-19 departments to those in other countries with different pandemic measures would be beneficial.

## Data Availability

Further data is available from the corresponding author upon reasonable request.
